# Lactate‐Induced Liquid–Liquid Phase Separation of HIF‐1α Drives Myeloid Cell Polarization and Immune Evasion in Colorectal Cancer

**DOI:** 10.1111/cpr.70212

**Published:** 2026-04-20

**Authors:** Chenhao Fu, Xiang Chen, Haoyu Huang, Lipeng Zhang, Haonan Huang, Fan Jiang, Fayang Lei, Honglong Yu, Jibiao Liu, Houping Zhang, Kan Dai, Zhixiong Wu, Huizi Li, Xi‐jian Dai, Shengxun Mao

**Affiliations:** ^1^ Department of Gastrointestinal Surgery, the Second Affiliated Hospital, Jiangxi Medical College, Nanchang University Nanchang Jiangxi China; ^2^ The First Clinical Medical College, Jiangxi Medical College, Nanchang University Nanchang Jiangxi China; ^3^ Department of Radiology, The Second Affiliated Hospital, Jiangxi Medical College, Nanchang University Nanchang China; ^4^ Jiangxi Provincial Key Laboratory of Intelligent Medical Imaging Nanchang China

## Abstract

Lactate‐Induced Liquid‐Liquid Phase Separation of HIF‐1α Drives Myeloid Cell Polarization and Immune Evasion in Colorectal Cancer.

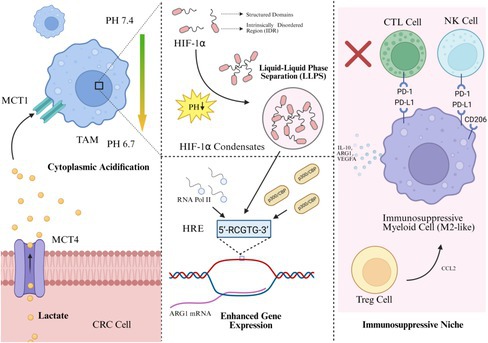

To the Editor,

Liquid–liquid phase separation (LLPS) is a key mechanism organizing biomolecular condensates for transcriptional regulation. In colorectal cancer (CRC), the immunosuppressive tumour microenvironment (TME), rich in lactate and dominated by myeloid cells, remains a therapeutic hurdle. A central unresolved question is how this metabolic perturbation, specifically high lactate, establishes fixed immunosuppressive transcriptional programs in immune cells.

While lactate is known to stabilise HIF‐1α, and HIF‐1α has inherent LLPS potential, a direct causal link is missing. Critical evidence gaps include whether lactate directly acts as a biophysical trigger for HIF‐1α LLPS; whether this LLPS is essential for amplifying HIF‐1α's transcriptional activity; and how it specifically reprogrammes myeloid cell transcription.

We propose a novel mechanism to address these gaps: lactate‐induced LLPS of HIF‐1α. We hypothesize that in the CRC TME, lactate directly promotes HIF‐1α coacervation. This forms a transcriptional condensate that potently and specifically amplifies genes driving myeloid cells into an immunosuppressive state, positioning LLPS as the central amplifier in this pathogenic axis (Figure 1).

**FIGURE 1:**
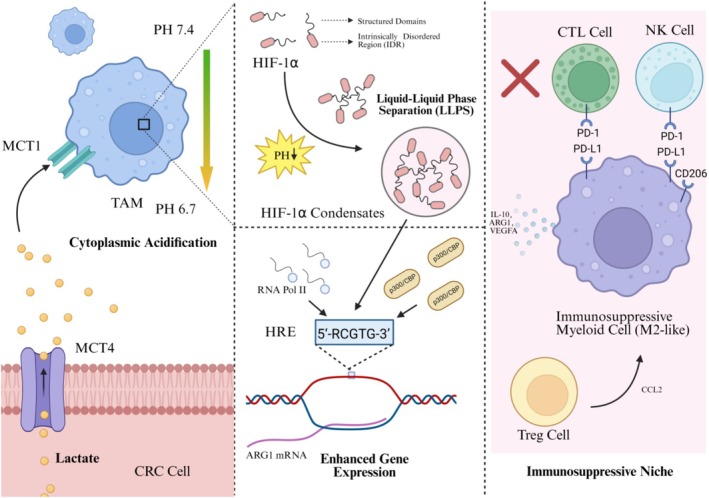
Proposed mechanisms of HIF‐1α LLPS‐driven myeloid cell reprogramming within the immunosuppressive network.

## 1 The Molecular Basis of HIF‐1α LLPS in CRC

We hypothesise that the accumulation of lactate in the tumour microenvironment of CRC triggers a transcriptional cascade that occurs not only due to conventional signal transduction pathways but also as a consequence of the modification of the fundamental biophysics of HIF‐1α to induce the generation of biomolecular condensates due to LLPS. This can be interpreted based on the following two primary molecular events.

### 1.1 Lactate as an Inducer of Phase Separation

Lactate is transported into myeloid cells via monocarboxylate transporters (MCTs) and is well‐established as a metabolic substrate and signalling molecule within the tumour microenvironment [1]. This established role provides the foundational context for our investigation.

Building upon this, we propose a novel speculative mechanism: lactate may specifically induce the liquid–liquid phase separation (LLPS) of HIF‐1α. We hypothesise that lactate import could lower cytoplasmic pH, and that this resultant acidification acts as a crucial biophysical trigger by altering the charge properties of HIF‐1α's intrinsically disordered regions (IDRs) to promote LLPS. Alternatively, or concurrently, lactate or a derivative such as lactoyl‐lysine might directly interact with HIF‐1α to facilitate the multivalent interactions necessary for phase separation. However, for HIF‐1α to function as a transcription factor, any cytosolic trigger must ultimately promote condensation in the nucleus. Thus, we acknowledge a key mechanistic gap: how cytosolic acidification links to nuclear HIF‐1α condensation. We propose that acidification may rapidly equilibrate across the nuclear envelope or pre‐condition HIF‐1α in the cytosol to lower its LLPS barrier in the nucleus. Direct validation will employ live‐cell imaging with nuclear‐targeted pH sensors to correlate nuclear acidification with HIF‐1α condensate formation. This will be complemented by nuclear fractionation to assess lactate levels, and by fluorescence recovery after photobleaching (FRAP) assays, a technique that measures molecular dynamics, on nuclear foci to confirm their liquid‐like properties characteristic of LLPS.

To test the aforementioned hypotheses and the critical spatial link between cytosol and nucleus, a combination of in vitro and live‐cell methods will be employed. First, in vitro reconstitution assays using purified HIF‐1α protein will be conducted in buffers of precisely tuned pH. This will determine if mildly acidic conditions (pH 6.5–6.8), mimicking lactate‐induced acidification, significantly enhance HIF‐1α condensate formation compared to physiological pH. To address the concern of distinguishing specific LLPS from non‐specific aggregation, our in vitro reconstitution strategy will employ rigorous validation. This includes demonstrating liquid‐like dynamics via FRAP, establishing concentration‐dependence, and using IDR‐disrupted HIF‐1α mutants as critical negative controls. Crucially, in vitro findings will be correlated with live‐cell condensate formation to ensure biological relevance. Second, live‐cell imaging of myeloid cells co‐expressing fluorescent HIF‐1α and a pH‐sensitive reporter, such as pH luorin (a genetically encoded fluorescent protein whose signal varies with pH), will assess whether cytoplasmic acidification upon lactate exposure is spatiotemporally coupled with HIF‐1α condensate formation. Third, biophysical methods, such as nuclear magnetic resonance (NMR) spectroscopy, will be used to investigate the theoretical premise that lactate or its derivative (e.g., lactoyl‐lysine) can directly interact with HIF‐1α, potentially acting as a molecular scaffold or client that promotes multivalency and LLPS.

Beyond establishing the occurrence of lactate‐induced HIF‐1α LLPS, a central question is whether this phase separation is a necessary mechanistic step. To address this, our experimental design includes key genetic perturbations. We will engineer LLPS‐deficient HIF‐1α mutants (e.g., through IDR disruption). The critical prediction of our model is that in myeloid cells expressing these mutants, lactate exposure will fail to induce the specific and potent upregulation of the immunosuppressive gene program (e.g., *ARG1, VEGFA*) despite normal HIF‐1α protein stabilisation. Conversely, artificially inducing HIF‐1α condensation in the absence of lactate should partially recapitulate the immunosuppressive phenotype.

To decisively attribute observed effects to lactate specifically, and not to general cellular acidification, our experimental design incorporates key controls: (1) Comparison of sodium L‐lactate with a non‐metabolizable or poorly metabolizable analog (e.g., sodium D‐lactate) and pH‐matched ionic controls (e.g., NaCl) will isolate the effect of the lactate anion. (2) The necessity of lactate transport will be tested using MCT1 inhibitors; subsequently, artificial cytoplasmic acidification will be attempted in these inhibited cells to see if pH drop alone can ‘rescue’ HIF‐1α LLPS. (3) Intracellular pH‐clamping techniques will be employed during lactate exposure to uncouple the presence of lactate from a decrease in cytoplasmic pH.

### 1.2 Transcriptional Amplification via HIF‐1α Condensates

The nucleation of HIF‐1α condensates is a novel concept that, in essence, is a departure from traditional understandings of HIF‐1α activation. The main hypothesis is that, in addition to protein stabilization, HIF‐1α LLPS is a major amplification tool that creates a condensate with high efficacy as a transcriptional platform. The condensate is expected to functionally concentrate transcriptional co‐factors such as p300/CBP, Mediator complex subunits, or RNA Polymerase II [2]. For functional validation of this enrichment, FRAP studies using fluorescently tagged p300, within HIF‐1α condensates, can be done to measure diffusion; this would reflect engagement or association. Also, this process is expected to strongly and specifically enhance genes responsible for myeloid polarization, such as *ARG1, VEGFA*, or *IL10*. Validation will include constructing a mutant form of HIF‐1α deficient in LLPS, probably through disruption within the IDR, that, while remaining stable, is significantly compromised in enhancing their expression as a specific target consequence of HIF‐1α LLPS.

## 2 LLPS‐Driven Immune Evasion via Myeloid Cell Reprogramming

We propose that the primary consequence of lactate‐induced HIF‐1α LLPS is the functional reprogramming of myeloid cells. This proposed mechanism is posited as a crucial driver of the immunosuppressive niche in CRC. This section explains the implications of the aforementioned biological mechanism.

### 2.1 Orchestration of Myeloid Cell Functional Polarization

The assembly of HIF‐1α biomolecular condensates creates a highly effective transcriptional platform for the coordinated induction of a group of genes known to contribute to immunosuppressive polarization [3]. The phenomenon of LLPS exceeds simple enhancement of gene expression levels, allowing for the coordinated engagement of multiple gene expression programs that distinguish the functional state of tumour‐associated myeloid cells. These include the simultaneous upregulation of *ARG1* and *NOS2*—genes typically associated with distinct functional programs but, in this context, convergently suppressing T cell function through arginine depletion and nitric oxide production, respectively. This is accompanied by the expression of *IL‐10* and *VEGFA* for suppressing adaptive immunity and supporting angiogenesis, and the enhanced expression of the chemokine receptor *CCR2* and its ligand *CCL2* to facilitate myeloid cell retention and recruitment, along with Tregs, into the tumour microenvironment [4] (Table 1).

**TABLE 1:** HIF‐1α LLPS‐driven myeloid cell reprogramming and the immunosuppressive network.

Functional module	Key target genes enhanced by HIF‐1α LLPS	Impact on immune cells	Immunosuppressive outcome
Metabolic dysregulation	*ARG1*(Arginase 1)	Depletes extracellular L‐arginine, impairing T cell receptor signalling and proliferation	Functional exhaustion of cytotoxic T lymphocytes (CTLs) within the tumour microenvironment (TME)
	*IDO1* (Indoleamine 2,3‐dioxygenase 1)	Depletes tryptophan and produces kynurenines, suppressing effector T cell activity and promoting Treg differentiation	Inhibition of antigen‐specific T cell responses and expansion of immunosuppressive regulatory T cells (Tregs)
	*NOS2* (Nitric Oxide Synthase 2)	Produces nitric oxide (NO), which suppresses T cell function	Contributes to the functional exhaustion of cytotoxic T lymphocytes (CTLs) within the TME
Immunosuppression and angiogenesis	*IL10* (Interleukin 10)	Suppresses antigen presentation and pro‐inflammatory cytokine production in macrophages and dendritic cells (DCs)	Attenuated activation and function of tumour‐infiltrating lymphocytes
	*VEGFA* (Vascular Endothelial Growth Factor A)	Induces abnormal tumour vasculature, creating a physical barrier for T cell infiltration and exacerbating hypoxia	Reduced infiltration of cytotoxic immune cells and perpetuation of an immunosuppressive, hypoxic TME
Myeloid cell recruitment and retention	*CCL2* (C‐C Motif Chemokine Ligand 2)	Recruits *CCR2+* monocytes from the circulation to the tumour site, serving as a precursor for tumour‐associated macrophages (TAMs) and myeloid‐derived suppressor cells (MDSCs)	Expansion of the immunosuppressive myeloid cell population, reinforcing the immunosuppressive niche
	*CCR2* (C‐C Motif Chemokine Receptor 2)	Promotes the retention of myeloid cells within the tumour tissue by enhancing responsiveness to *CCL2*	Sustained maintenance and stability of the immunosuppressive network

### 2.2 Sculpting an Immunosuppressive Microenvironment and Clinical Progression

The myeloid cell functional polarization builds a strong barrier for anti‐tumour immunity [5, 6]. The LLPS‐driven amplification of transcriptional expression results in the establishment of the immunosuppressive microenvironment by several distinct and cumulative mechanisms: metabolic dysregulation (such as the loss of arginine and tryptophan), the production of anti‐inflammatory cytokines and the stimulation of the immune checkpoint pathways (such as PD‐L1/PD‐1). This micro‐environment negatively inhibits cytotoxic T lymphocytes and natural killer cells on one hand; on the other hand, it actively accumulates Tregs [7]. The link between this micro‐environment and clinical progression within CRC is clear; this micro‐environment also builds resistance to therapy generally and immune checkpoint therapy specifically and promotes metastasis [8, 9]. For these reasons, while direct spatial evidence linking lactate‐rich niches to HIF‐1α condensates in human CRC is lacking, strong indirect clinical evidence supports the relevance of this proposed axis: HIF‐1α is an established poor‐prognosis factor; oncogenic drivers like KRAS can create the lactate‐rich TME; and myeloid infiltration is linked to immunosuppression and therapy resistance [10]. Therefore, if the aforementioned validation confirms that HIF‐1α LLPS is a necessary amplifier downstream of lactate signalling, then targeting the lactate/HIF‐1α LLPS axis would represent a promising, hypothesis‐driven therapeutic strategy to reverse the immunosuppressive microenvironment. Its therapeutic potential is precisely predicated on disrupting this specific, potent amplification node that may not be redundant with other lactate‐ or HIF‐1α‐mediated pathways.

## 3 Conclusion and Therapeutic Perspectives

In short, this letter portrays lactate‐induced LLPS of HIF‐1α as the transformative mechanism paradigm for immune evasion in colorectal cancer based on the proposed model that considers biomolecular condensation as the amplifier for the mechanistic amplifier of a key metabolic signal for the stable, immunosuppressive transcriptional outcome in myeloid cells, connecting metabolic responses to immunodulatory decision goals. Abnormalities of normal transcriptional regulation, according to this proposed model, are suggested for the direct pathological remodelling of the tumour microenvironment.

This model points to a novel and speculative therapeutic target. The LLPS axis has characteristic fragilities in addition to those of traditional pathways. Future investigations should therefore test the possibility of identifying small‐molecule condensate modulator compounds that can selectively disrupt HIF‐1α condensates without affecting their basal expression or stability, hence specifically counteracting the pathologic potentiation of HIF‐1α activity. To address the pathologic potentiation of HIF‐1α activity, LLPS inhibitors could be incorporated into combination regimens; these compounds could potentially cooperate with current immunotherapeutic regimens, including immune checkpoint inhibitors in the setting of myeloid‐dense CRC tumours, where resistance has often been encountered. Finally, upstream therapies, including MCT1 inhibitors targeting lactate transport, provide a tangible way to counter the initial insult triggering this harmful LLPS process.

While this conceptual model is derived from mechanistic insights, its clinical relevance requires validation in human CRC. We plan to investigate whether punctate HIF‐1α staining (indicative of condensates) in patient samples spatially correlates with high lactate metabolism (e.g., LDHA*/MCT1* expression), immunosuppressive myeloid markers (e.g., *ARG1, CD163*) and adverse clinical outcomes. This correlative analysis in tissue microarrays will be a critical step in evaluating HIF‐1α LLPS as a potential prognostic biomarker and therapeutic target.

Looking forward, there is an urgent need to leverage these concepts to translationally validate the existence and importance of HIF‐1α condensates in human CRC samples, potentially providing new biomarkers. The development of high‐throughput platforms that can provide chemical probes targeting LLPS is of prime importance. Finally, targeting the lactate and HIF‐1α LLPS module holds great promise to disrupt the immunosuppressive network of CRC and open new therapeutic perspectives to restore anti‐tumoral immune responses.

## Author Contributions

**Chenhao Fu:** writing – original draft, Writing – review and editing, software, methodology. **Xiang Chen:** writing – original draft, visualization. **Haoyu Huang:** writing – original draft, visualization, software. **Lipeng Zhang, Haonan Huang:** writing – original draft, software. **Fan Jiang, Fayang Lei, Honglong Yu, Jibiao Liu, Houping Zhang:** writing – original draft, software. **Kan Dai, Zhixiong Wu:** data curation, conceptualization. **Huizi Li, Xi‐jian Dai:** funding acquisition, project administration. **Shengxun Mao:** writing – review and editing, visualization, validation, supervision, funding acquisition and project administration.

## Funding

This work was supported by the internal funding project of Nanchang University (Grant Number: 2023efyBo5), National Natural Science Foundation of China (grant nos. 82572200, 82460341), Science Foundation for Distinguished Young Scholars of Jiangxi Province (grant no. 20242BAB23086), GanPo Talent plan of Jiangxi Province (grant no. gpyc20240213), Jiangxi Province Higher Education Association (grant no. ZN‐D‐014), Research Project on Educational Reform for Postgraduate Students at Nanchang University (grant no. JXYJG‐2025‐063).

## Ethics Statement

The authors have nothing to report.

## Conflicts of Interest

The authors declare no conflicts of interest.

## Data Availability

Data sharing not applicable to this article as no datasets were generated or analysed during the current study.
